# Epigenetic Therapy Promotes the Ratio of Th1/Th17 Lineage to Reverse Immune Evasion and Treat Leukemia Relapse Post-allogeneic Stem Cell Transplantation in Non-APL AML Patients

**DOI:** 10.3389/fmolb.2020.595395

**Published:** 2021-08-24

**Authors:** Yang Xi, Dai Jingying, Li Chenglong, Zheng Hong, Zhang Rong, Wang Xiaodong, Wang Chunsen, Huang Xiaobing

**Affiliations:** ^1^Sichuan Provincial People's Hospital, Affiliated Hospital of University of Electronic Science and Technology of China, Chengdu, China; ^2^Penn State Cancer Institute, Penn State University College of Medicine, Hershey, PA, United States

**Keywords:** epigenetic regimen, non-APL AML, transplant relapse, immune evasion, Th1/Th17

## Abstract

To reverse the early-stage relapse post-hematopoietic stem cell transplantation, we investigated the safety and efficacy of a new epigenetic regimen (chidamide and decitabine plus thymalfasin simultaneously) on acute myeloid leukemia patients (excluding acute promyelocytic leukemia). Twenty-four patients were enrolled in this observational study during April 2015 to May 2018. The most common adverse event was reversible CTCAE grade 2 thrombocytopenia (20/24). Strikingly, all 24 patients had response to this epigenetic regimen accompanied with decreased measurable residual disease. The overall survival rate is 79.2% (19/24), with a relapse-free survival rate of 79.2% (19/24). During this regimen treatment, Th1 cells and CD3+CD4-CD8+T cells increased, and Th17 cells decreased gradually. The status of high Th1 and low Th17 cells was still observed on the 3rd month after discontinuation of this regimen. Interestingly, the significantly elevated ratio of Th1/Th17 seemed to reflect the treatment-related immune effect, which may be a valuable marker to be monitored in the early-relapse stage for evaluating the efficacy and prognosis.

## Introduction

Acute myeloid leukemia (AML) is a heterogeneous disorder with high morbidity and mortality. According to the prognosis assessment by different characters in morphology, immunophenotype, cytogenetics, and molecular biology, the patients were divided into a low-risk group, an intermediate-risk group, and a high-risk group (Estey, 2018). Allogeneic hematopoietic stem cell transplantation (allo-HSCT) is an effective, and sometimes the only, curative post-remission therapy for AML patients. Based on genetic risk classification, the published data have suggested that allo-HSCT may be recommended for high-risk and most intermediate-risk AML but not for low-risk AML in first complete remission (CR1) (Takami, 2018). Recently, the role of allo-SCT in low-risk AML in CR1 is being established with the development of a risk-directed, measurable residual disease (MRD)-based strategy (Döhner et al., 2017). Acute promyelocytic leukemia (APL) patients in the first CR are expected to have 70–80% disease-free survival; thus, HSCT is not recommended in the first CR (Takami, 2018). So, allo-HSCT can significantly improve the overall survival (OS) of AML except APL. However, a part of this patient population still experience relapse, which leads to a poor long-term outcome (Thanarajasingam et al., 2013; Lee et al., 2019; Rautenberg et al., 2019). The EBMT registry data of 8,162 adult AML patients who relapsed between 2000 and 2018 after allo-HCT showed that the 2-year OS from relapse was 17% (Bazarbachi et al., 2020). At present, disease relapse remains the most important cause for failure post-allo-HSCT. Pre-emptive intervention, when MRD turns positive again, may be an effective approach to avoid hematological relapse (HR) (Schroeder et al., 2018). Current treatment strategies for relapse post-transplant mainly include reduction or discontinuation of immunosuppressant, donor lymphocyte infusion (DLI), chemotherapy, hypomethylating agents (HMAs), and second allo-HSCT(Yan et al., 2012; Craddock et al., 2016a; Tsirigotis et al., 2016; Wouters and Delwel, 2016; Schroeder et al., 2018). However, the clinical efficacy of these approaches is far from satisfactory. Therefore, a new treatment strategy still needs to be developed.

Rapid and effective promotion of immune reconstitution after HSCT can not only induce immune tolerance and prevent graft-*vs*.-host disease (GVHD) but also maintain strong graft-*vs*.-leukemia (GVL) effect to prevent relapse after transplantation. Epigenetic modifications, such as DNA methylation and histone and non-histone acetylation, are important regulators of various aspects in the life of T cells, including development, activation, and differentiation into effector T cells and T cell immunity. On the one hand, histone acetylation is considered as a crucial role in these processes. Histone deacetylase inhibitors (HDACis) were demonstrated *in vitro* as crucial regulatory functions for histone acetylation (HDAC) during the activation and differentiation of T helper (Th) cells, which are due to chromatin-mediated effects as well as the impaired activities of key regulatory factors (Avni et al., 2002; Boucheron et al., 2014; Ellmeier and Seiser, 2018). Inhibition of HDAC1 and HDAC2 induces the appearance of MHC class II-restricted CD4+ T cells with upregulated CD8+ T cell lineage genes and a CD8+ T cell effector program under homeostatic conditions that was further enhanced upon activation under Th0 cell and Th1 cell (but not Th2 cell) conditions (Boucheron et al., 2014). Orly Avni et al. (2002) showed that T cell receptor stimulation results in the early activation of the genes, encoding both interleukin 4 and interferon-γ, and then establishes a selective pattern of histone acetylation, which may further lead to the activation of CD4+ T cells fixing to Th1 lineage identity. Interestingly, HDAC family proteins can also regulate Th17 cells and Th17 cell-driven autoimmunity. SIRT1 is highly expressed in TH17 cells compared with other Th cell subsets. Limagne et al. (2017) found that SIRT1-deficient CD4+T cells show a reduction in Th17 cell differentiation and SIRT1 agonists block Th17 cell differentiation by deacetylating STAT3. A latest research also confirmed that chidamide, a new HDAC inhibitor, markedly downregulated histone deacetylase SIRT1, except when inhibiting HDAC1 and HDAC2 (Xu et al., 2020).

On the other hand, the roles of DNA methyltransferases (DNMT) and DNA methylation in T lymphocytes have been extensively reviewed (Wilson et al., 2009; Leoni et al., 2015). Gamper et al. (2009) found that loss of DNMT3a in CD4+T lymphocytes resulted in increased and dysregulated cytokine expression. Differentiated Th2 cells lacking DNMT3a could easily induce IFN-γ production once placed in Th1 conditions, whereas wild-type cells remained much more stable in their lineage decision. These results highlighted a role for DNA methylation and histone acetylation not only in the regulation of cytokine expression but also in the stabilization of T cell phenotypes (especially in the fixation of Th1 lineage identity). In addition, thymus function is also an important factor affecting the development and differentiation of T cells. Decreasing and degraded thymus function is closely related to T-cell development, differentiation, and maturation, especially in the long recovery period of the immune system after HSCT. Thymalfasin (thymosin-alpha 1) is an immunomodulating agent, which is reported to be able to enhance the Th1 immune response by increasing the production of interferon-γ, IL-2, and IL-3 and the expression of the IL-2 receptor (Sjogren, 2004; He et al., 2017).

In the present study, we investigated the safety and efficacy of a new epigenetic regimen (HMA–decitabine and HDACi–chidamide plus thymalfasin) for non-APL AML patients who had molecular relapse after HSCT. The majority of patients in this cohort achieved complete molecular remission (CMR) again and long relapse-free survival (RFS) after this regimen. Interestingly, the trend in Th1/Th17 ratio seems to indicate early molecular relapse and therapeutic efficacy.

## Methods

### Patients, Treatment, and Samples

A total of 24 non-APL AML patients were enrolled in this study. All patients needed multiple induced chemotherapy to reach complete hematologic remission (CHR) or CMR and experienced relapse before allo-HSCT. Although considered as high-risk patients, they all achieved CHR at the time point of allo-HSCT, including three patients with detectable MRD, and consecutively received allo-HSCT at our institute from April 2015 to May 2018.

### Transplantations

Transplantations were performed as our convention as listed below. Eighteen recipients of human leukocyte antigen (HLA)-haploidentical related transplantation and six recipients of HLA-matched related transplantation were conditioned with cytarabine (4.0 g/m^2^ per day for HLA-haploidentical transplantation or 2.0 g/m^2^ per day for HLA-matched transplantation, days −10 to −9), fludarabine (30 mg/kg per day, intravenously, days −10 to −6), busulfan (3.2 mg/kg per day, intravenously, days −8 to −6), cyclophosphamide (1.4–1.6 g/m^2^ per day, days −5 to −4), and rabbit ATG-F (Fresenius, Biotech GmbH, Munich, Germany, 5 mg/kg per day for HLA-haploidentical and unrelated donor transplantation or 2.5 mg/kg per day for HLA-matched transplantation, intravenously, days −5 to −2). All subjects received G-CSF-mobilized bone marrow (BM) and blood cells. After transplantation, they received cyclosporine, short-term methotrexate, and anti-CD25 monoclonal antibody (basiliximab) for GVHD prophylaxis.

### Ethics Approval

The study was approved by the Ethics Committee of the Affiliated Hospital of the University of Electronic Science and Technology of China [ethical batch number: *2015kelun(16)*], and all patients or their guardians provided written informed consent to participate in the study in accordance with the Declaration of Helsinki. In addition, this study was also registered at http://www.chictr.org.cn/index.aspx (Chinese Clinical Trial Registry, ChiCTR) as ChiCTR2000032330.

### The Epigenetic Regimen

Once leukemia relapse was confirmed by turning positive again on MRD, the epigenetic regimen in this study was administrated. The detailed prescription of the three drugs was as follows: (1) chidamide, orally at 0.5 mg/kg twice a week for children or 10 mg for 6 days per week for adults, (2) decitabine, hypodermic injection at 10 mg for 2 days per week, (3) thymalfasin, hypodermic injection at 1.6 mg twice a week, and (4) 4 weeks was considered as a cycle and the treatment plans contained three cycles at least. If morphologic evaluations and the quantitative measurement of MRD in BM samples showed signs of relapse, the immune monitoring and the treatment with this regimen started. The scheduled time points for immune monitoring were 0, 1, 2, and 3 months after the start of this regimen and the 3rd month after discontinuation. Epigenetic treatment would go on for 2 months when MRD turned negative quickly. In contrast, modified DLI regimen will be given to combine with this regimen until achieving CMR if MRD still increased in two successive analysis or turned positive again during treatment. The cutoff date for follow-up was May 20, 2020. The median follow-up time after post-transplantation relapse was 29 (4–59) months in the entire cohort.

### Clinical Definition

These patients were diagnosed with the use of the WHO 2008 consensus criteria and stratified according to the NCCN 2016 guideline and treatment response. The MRD schedules were planned in advance, which included detecting residual leukemia cells by flow cytometry (FCM), leukemia fusion gene by qRT-PCR, and prognostic gene of initial diagnosis by qRT-PCR. Allo-HSCT was performed on high-risk patients, and the clinical efficacy was evaluated as follows: overall response rate (ORR) was defined as a decrease in MRD; CMR was defined as a negative turn of MRD level; and partial response, stable disease, and progression of disease were respectively defined as a decrease in MRD, no significant change in MRD, and an increase in MRD with hematologic relapse.

### Laboratory Analysis

#### Measurement of MRD From Bone Marrow Specimens

All experimental operations were completed by the Kindstar Global Medical Special Inspection Group according to their standard experimental procedures. Leukemia fusion genes, such as AML1-ETO transcript level, were calculated as the percentage of fusion gene transcript copies/ABL copies using qRT-PCR. All of the samples with an undetectable fusion gene transcript had ≥12,500 copies of ABL to guarantee that at least a 4-log reduction of fusion gene transcript levels (0.04%) could be detected. What is more, the evaluation of the proportion and immunophenotype of primitive cells was analyzed through FCM.

#### Detection of Immune Cells by Flow Cytometry

Protocol-specified immunophenotypic analysis was performed at baseline for all patients and 24 healthy controls and for relapse patients at the time points of 0, 1, 2, and 3 months after the start of this regimen and the 3rd month after discontinuation. CTL cells were defined as CD3^+^CD4^−^CD8^+^, natural killer (NK) cells as CD3-CD56^+^, T help (Th) cells as CD3^+^CD4^+^CD8^−^, B cells as CD3^−^CD19^+^, Th1 cells as CD3^+^CD4^+^CD8^−^INFγ^+^, Th2 cells as CD3^+^CD4^+^CD8^−^IL4^+^, Th17 cells as CD3^+^CD4^+^CD8^−^IL17^+^, and Treg cells as CD3^+^CD4^+^CD25^+^Foxp3^+^. All experimental operations were completed by Kindstar Global Medical Special Inspection Group according to their standard experimental procedures.

### Data Analysis and Statistics

Statistical analysis of the data was performed using GraphPad Prism, version 5.0, for Macintosh (GraphPad Software, San Diego, CA). The data were presented as mean ± SEM. Student's unpaired *t*-test (two-tailed) was used for the statistical analysis of two groups. Differences between groups were analyzed using SPSS (New York, NY, USA). A *P*-value <0.05 was considered as statistically significant.

## Results

### Patients' Characteristics

Twenty-four patients were enrolled into this study. All patients were diagnosed with non-APL AML as per WHO classification and underwent allo-HSCT due to the high-risk features of their disease. Risk stratification was determined as per the 2016 NCCN guideline. Specifically, patients who have persistent positive MRD and experienced irreversible relapse before transplantation were defined as high risk. Patient characteristics at the time of enrollment are shown in Table 1. The median age was 25 years old (range, 7–45). Among the 24 enrolled patients, four patients did not achieve CMR prior to transplantation. The patients were monitored for MRD by FCM and real-time PCR. All patients enrolled had MRD relapse post-transplantation. Administration of this epigenetic regimen was initiated when MRD turned positive. The median time of MRD relapse after allo-HSCT was 75 days (range, 30–120) in 20 patients who had negative MRD post-transplantation initially but turned positive later. The other patients had persistent positive MRD after transplantation. All patients received the regimen for at least 3 months. Prior to the treatment, tapering and discontinuation of immune suppressors were applied to all patients. Two patients received DLI due to persistent MRD after the above-mentioned two treatments.

**Table 1:** Characteristics of patients and donors.

**Characteristics**	***n* = 24**
Age in years, median (range) of the recipient	29 (6–52)
Gender, number	
Male	10
Female	14
White blood cells at diagnosis × 10^9^/L, median (range)	37 (18–95)
Immunophenotype	
M1	2
M2	10
M4	6
M5	6
Poor prognostic gene mutations, number	
Positive	20
Negative	3
Unknown	1
Poor karyotype, number	
Normal	9
Sole t (8: 21)	5
Complex karyotype	10
Disease status, number	
First CR (CR1)	18
Second CR (CR2)	6
Courses required to achieve CR	
1	14
>1 (including CR2)	10
Time from diagnosis to transplant in months, median (range)	6 (4–19)
Donor source	
Haploidentical	12
Human leukocyte antigen-matched sibling	6
Unrelated donor	6
Stem cell source	
G-CSF mobilized BM+peripheral blood	12
G-CSF mobilized peripheral blood	12
Median CD34+ count, × 10^6^/kg (range)	2.9 (1.8–8.5)
Median total nucleated cell count, × 10^8^/kg (range)	9.8 (6.1–18.4)

### This Epigenetic Regimen Was Safe and Tolerable in Patients With Non-apl AML Post-transplantation

None of the patients had severe adverse events requiring discontinuation during this epigenetic regimen (Table 2). The most common adverse event was reversible CTCAE grade 2 thrombocytopenia (the platelet counts were all above 20 × 10^9^/L), without apparent bleeding symptoms. Other adverse events that were possibly or probably related to the treatment include grade 1 constitutional symptoms (24/24), reversible grade 2 transaminitis (9/24), grade 1 renal dysfunction (4/24), grade 1 digestive tract symptoms (13/24), grade 2 neutropenia (6/24), and grade 1 skin rash (12/24). No GVHD was observed in patients who received this regimen as monotherapy, whereas two patients combining with DLI developed grade 3 GVHD (skin and gastrointestinal tract).

**Table 2:** Adverse events from the epigenetic regimen.

**Variable**	**Number of patients**
Patients who could be evaluated	24
Grade 2 thrombocytopenia	24
Grade 1 constitutional symptoms (fever, malaise, fatigue)	24
Grade 2 transaminitis	9
Grade 1 digestive tract symptoms (nausea, vomiting, ventosity)	13
Grade 1 renal dysfunction	4
Grade 1 skin rash	12
Grade 2 neutropenia	6
Grade 3 pneumonia	0
Graft-vs.-host disease exacerbation	0

### The Majority of Patients Achieved Complete MRD Remission With This Regimen

Once started on this regimen, the MRD level was monitored every month. Strikingly, all 24 patients had responded to this regimen with decreased MRD (ORR 100%). The overall survival rate is 79.2% (19/24), with a relapse-free survival rate of 79.2% (19/24). Of note is the fact that, among the 24 patients who achieved CMR, 19 patients have durable response, with MRD remaining negative even at 3 months or longer beyond discontinuation of the treatment. Five patients had continuous positive MRD and finally died due to leukemia relapse. The clinical outcome of each patient is shown in Table 3.

**Table 3:** Clinical outcome of our epigenetic regimen therapy on these patients.

**Patients' ID**	**Time of MRD negative again from relapse (months)**	**During time of treatment (months)**	**Relapse-free survival (months)**	**Overall survival (months)**
1	1	3	58	59
2	2	4	56	58
3	1	3	52	53
4	1	3	50	51
5	1	3	47	48
6	2	4	45	47
7	3	5	41	44
8	1	3	41	42
9	1	3	37	38
10	4	4	0	5
11	6	6	0	7
12	2	4	33	35
13	4	6	27	31
14	7	9	22	29
15	1	3	26	27
16	5	5	0	6
17	2	4	27	29
18	3	5	24	27
19	2	4	27	29
20	2	2	0	4
21	2	4	23	24
22	1	3	25	26
23	8	8	0	8
24	1	3	24	25
Median	2	4	27	29

### Th17 Cells Were Increased in Patients With MRD Relapse

**Figure 1:**
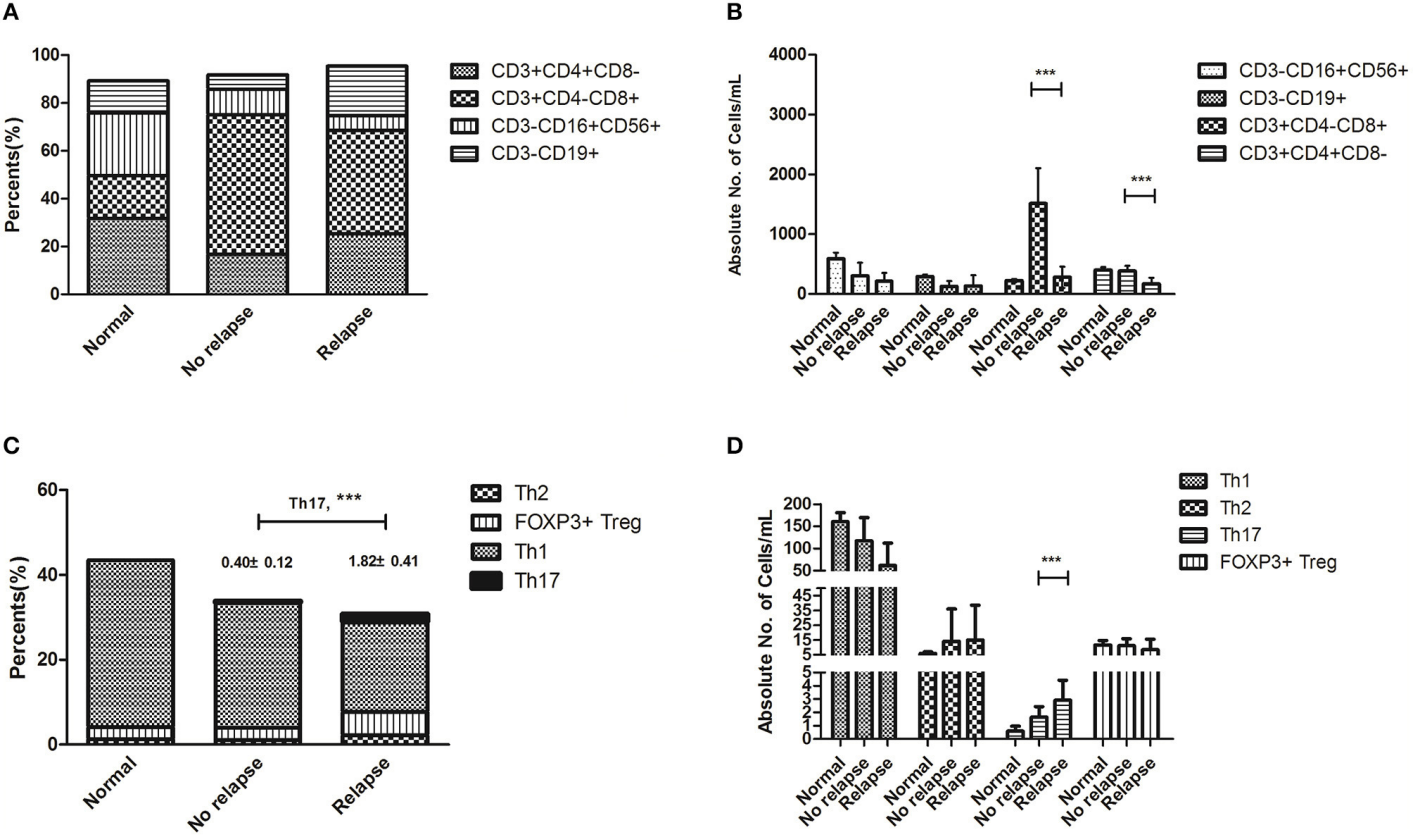
Immune status at the onset of relapse after hematopoietic stem cell transplantation (HSCT) compared with that of patients remaining at remission. Statistical differences are marked by the *P*-values. **(A)** The percentages showed no statistical differences in CTL, T helper, NK, and Treg cells. **(B)** It showed significantly decreased absolute counts of CD3^+^CD4^−^CD8^+^T cells (281.2 ± 174.1 vs. 1514.5 ± 576.6, *P* < 0.001) and CD3^+^CD4^+^CD8^−^ T cells (172.7 ± 100.7 vs. 389.7 ± 82.5, *P* < 0.001) in patients with relapse after HSCT. **(C)** There was a significant increase in the percentages of Th17 cells (1.82 ± 0.41 vs. 0.40 ± 0.12, *P* < 0.001) and no statistical differences in Th1, Th2, and FOXP3^+^ Treg cells. **(D)** There was a significant increase in the absolute count of Th17 cells (4.88 ± 2.46 vs. 1.50 ± 0.72, *P* = 0.001), while there were no statistical differences in Th1, Th2, and FOXP3^+^ Treg cells. ***P < 0.05.

**Figure 2:**
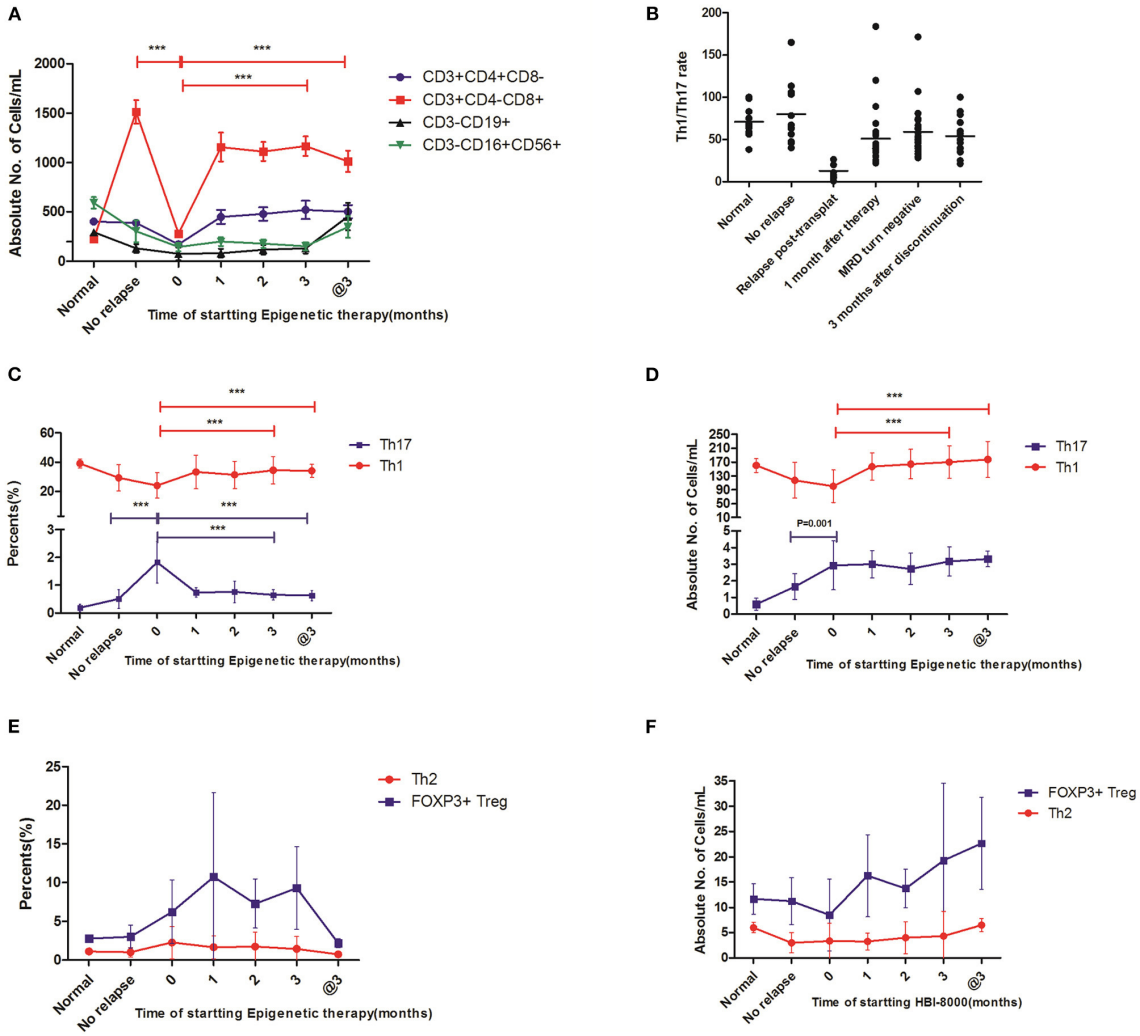
The immunological effect of this epigenetic regimen on these relapse patients post-transplantation. Statistical differences are marked by the *P*-values. @3, on the 3rd month after discontinuation of this regimen. **(A)** The changes of absolute counts in CTL, T helper, NK, and Treg cells showed significantly decreased absolute counts of CTL cells and T helper cells in the patients with relapse and also showed that CTL cells increased gradually after this epigenetic regimen, whereas no statistical differences were observed in T helper, NK, and Treg cells. **(B)** The Th1/Th17 ratio significantly decreased in patients with relapse and increased gradually after this epigenetic regimen. **(C,D)** The changes in Th1 and Th17 cells showed that both percentages and absolute counts of Th1 cells increased gradually after this epigenetic regimen. In contrast, Th17 cells decreased gradually in percentages, whereas there was no statistical difference in absolute counts. **(E,F)** The changes in Th2 and Treg cells in percentage and absolute counts showed no statistical differences after this epigenetic regimen ***P < 0.05.

We performed extensive correlative studies to investigate the mechanisms for the clinical impact of the epigenetic regimen in this cluster. We first evaluated the distribution of each immune component from these patients prior to this regimen, at which point all patients had MRD relapse. Samples from a cohort of 24 patients who remained CMR post-transplant were used as controls. We observed significantly decreased absolute counts of CD3^+^CD4^−^CD8^+^T cells (281.2 ± 174.1 vs. 1,514.5 ± 576.6, *P* < 0.001) and CD3^+^CD4^+^CD8^−^ T cells (172.7 ± 100.7 vs. 389.7 ± 82.5, *P* < 0.001) in the patients with relapse compared with those of patients remaining in remission, whereas NK cells and B cells showed no statistical difference (Figures 1B, 2A). The percentages showed no statistical differences in CTL, T helper, NK, and Treg cells (Figure 1A). Importantly, there was a significant increase in the percentage (1.82 ± 0.41 vs. 0.40 ± 0.12, *P* < 0.001) and absolute count (4.88 ± 2.46 vs. 1.50 ± 0.72, *P* = 0.001) of Th17 cells, while no difference of Th1, Th2, and FOXP3^+^ Treg cells was observed (Figures 1C,D). These data seemed to suggest that Th17 cells contributed to the relapse of non-APL AML patients after transplantation.

### The Epigenetic Regimen Promoted the Percentages of Th1 Cells and Decreased the Percentages of Th17 Cells

We next examined the level of Th1, Th2, and Th17 from the 24 relapse patients prior to this regimen and monthly in the following 3 months after this regimen. We found that the percentages (34.5 ± 9.43 vs. 21.1 ± 9.70%, *P* < 0.001) of Th1 cells increased gradually during the 3-month treatment (Figures 2C,D). In contrast, the percentages of Th17 cells (0.65 ± 0.19 vs. 1.82 ± 0.41%, *P* < 0.001) decreased (Figure 2C). Importantly, the status of high Th1 and low Th17 cells was still observed on the 3rd month after discontinuation of the regimen (Figures 2C,D). In addition, Th2 and Treg cells in percentages and absolute counts showed no statistical difference (Figures 2E,F). These important findings might contribute to the clinical efficacy of CMR.

### Enhanced Th1/Th17 Ratio Was Associated With the Downtrend of MRD

We further evaluated whether the trend in Th1/Th17 ratio was associated with the clinical outcome. To this end, we analyzed the correlation between Th1/Th17 ratio and MRD levels. Our results showed a significantly decreased Th1/Th17 ratio (12.9 ± 7.81 vs. 80.0 ± 37.9, *P* < 0.001) in patients with relapse compared with that of patients remaining in remission. Importantly, compared with the value at the time of relapse, the Th1/Th17 ratio significantly increased (58.9 ± 30.0 vs. 12.9 ± 7.81, *P* < 0.001) on the 3rd month post-treatment, and a high Th1/Th17 ratio was still maintained on the 3rd month after discontinuation (Figure 2B). Meanwhile, we confirmed that an enhanced Th1/Th17 ratio was associated with decreased MRD levels. According to the analysis for this cohort, we showed that Th1/Th17 ratio, a good biomarker, seemed to better reflect the immune effect than Th1/Th2 ratio and Th1 or Th17 alone in the follow-up time after this regimen.

## Discussion

According to the 2016 World Health Organization's criterion for AML patients, the AML patients were divided into a low-risk group, an intermediate-risk group, and a high-risk group. Conventional chemotherapeutic approaches, as the mainstay for AML treatment, usually brings CR in these patients of the intermediate-risk and the high-risk group but can fail in long-term leukemia control due to the high incidence of subsequent relapse (Cornelissen and Blaise, 2016). Allo-HSCT, which offers a strong GVL effect, is generally recommended as a post-remission therapy for AML patients who are eligible candidates (Cornelissen and Blaise, 2016; Pei and Huang, 2019). Results from previous studies suggested that allo-HSCT offers a significant advantage with regard to RFS and OS compared with that of chemotherapy or autologous transplantation particularly. However, post-transplantation relapse remains one of the most important causes of transplant failure, and further therapeutic options are limited. Quickly and effectively reconstructing the donors' immune system post-transplantation is an effective method to prevent a relapse. Based on the crucial role of epigenetic regulations on the immune system (Avni et al., 2002; Gamper et al., 2009; Wilson et al., 2009; Boucheron et al., 2014; Leoni et al., 2015; Wouters and Delwel, 2016; Limagne et al., 2017; Ellmeier and Seiser, 2018; Xu et al., 2020) as discussed in the “Introduction,” it is reasonable to consider that epigenetic regulation plays an important and unique role in promoting immune reconstitution and preventing leukemia relapse post-allo-HSCT. More importantly, these drugs have been widely used in the clinical practice of AML and acute lymphocytic leukemia by hematologists and oncologists.

In our research, we show that a novel epigenetic regimen based on HDACis combined with HMAs has eliminated MRD post-HSCT, which brings on the outcomes of CMR and potentially long survival, with an acceptable toxicity and no obvious GVHD in non-APL AML patients who have post-transplant relapse. Although limited by sample number, our research shows a promising clinical efficacy as manifested by high ORR and CMR. To our knowledge, this is the first clinical evidence for double-epigenetic therapy on non-APL AML patients with post-transplant relapse. At the same time, we also add thymalfasin to regulate thymic function. In addition, we demonstrate an obvious promotion for Th1 lineage identity and the significantly elevated ratio of Th1/Th17 in patients who received this regimen, which may contribute to the efficacy for reversing post-transplantation relapse. We also confirm that pre-emptive intervention, when MRD turns positive again, is an effective approach to avoid HR from the progression of molecular relapse.

Histone acetylation is critical in regulating gene expression for many immune processes (Marks et al., 2001), and recent evidence indicates that HDAC inhibitors are modulators of the immune system, providing guidance to using these drugs in tumor immunotherapy. In fact, HDAC inhibitors fall into five main classes based, in part, on their chemical structures and, in part, on their specificity. These include the following: (1) hydroxyamic acids, (2) cyclic tetrapeptides, (3) benzamides, (4) ketones, and (5) aliphatic acids. Differently from pan-HDAC inhibitors (such as SAHA), entinostat (MS-275) selectively inhibits class I HDACs (HDAC 1–3) and has been extensively studied for solid tumors, such as breast cancer (Knipstein and Gore, 2011; Trapani et al., 2017). Interestingly, chidamide, which targets HDAC1, 2, 3, and 10, also belongs to the benzamide class but exerts obvious anti-tumor immunity effect in relapsed and refractory peripheral T cell lymphoma and acute myeloid leukemia (Li et al., 2015; Chan et al., 2017). Nevertheless, a more notable case than targeting tumor cells is chidamide-mediated immunomodulatory effects. It was reported that chidamide induced MHC class I-like antigens (e.g., MICA, MICB, etc.) and the ligand of the natural killer receptor (NKG2D) on *in vitro* cell lines and in clinical samples (Schmudde et al., 2008). Chidamide also enhances the cytotoxic effects of human peripheral mononuclear cells *ex vivo* on K562 target cells *via* up-regulating NKG2D and granzymes to activate NK cells (Ning et al., 2012). What is more, chidamide could induce the expression of the leukemia-specific antigen PRAME (preferentially expressed antigen in melanoma) in both cell lines and leukemia blasts from patients, resulting in increased PRAME-specific and CTL-mediated cytotoxicity against leukemia *in vitro* (Yao et al., 2013). Most of these researches were performed using cell lines *in vitro*; clinical researches were rare. Prior to this research, we treated several patients who suffered from post-transplantation relapse with chidamide monotherapy and achieved good results, but our clinical practice from patients post-transplantation showed that chidamide tended to promote the lineage identity of T cell subsets, especially Th1 augmentation, and then increased CTL cells [type 1 immunological response according to Gérard Eberl's definition about immunity by equilibrium (Eberl, 2016)].

Mutations in DNMT3A, TET2, ASXL1, IDH1, and IDH2 are recurrently found in AML and contribute directly or indirectly to alterations in DNA methylation (Lindblad et al., 2017). Figueroa et al. identified a signature of recurrent hypermethylation of 16 genes across AML genetic subtypes and confirmed that classification of AML patients by DNA methylation profile is an independent predictor of clinical outcome (Figueroa et al., 2010). Hypomethylating agents are cytidine analogs that incorporate during DNA synthesis. Substitution for nitrogen at position 5 of the pyrimidine ring in place of carbon impedes methylation of the base by DNMT. Currently, decitabine (5-aza-2-deoxycitidine) and azacitidine (5-azacitidine) are FDA-approved for the treatment of patients with MDS and older AML patients unlikely to tolerate induction chemotherapy or for those that have relapsed post-HSCT. The mutation-associated AML described above may influence not only AML cells but also immune cell subsets, with the rapid development of immunotherapy in recent years. NK cells are modulated by hypomethylating agents, with the expression changes of inhibitory and activating receptors on the cell surface, including an increased expression of KIR proteins and a decreased expression of the NKG2D activating receptor. For example, promoter methylation of KIR genes has been shown to consistently suppress KIR expression on NK cells, whereas hypomethylation enhances KIR expression (Chan et al., 2003). Within T cell subsets, methylation play a critical role for regulating the expression of immune checkpoint inhibitors (such as PD-1, CTLA4, and TIGIT), co-stimulatory molecules including CD28 and CD80, and FOXP3 (Lindblad et al., 2017). For example, azacitidine made the PD-1 promoter hypomethylation in CD8+ T-cells from AML and MDS patients (Ørskov et al., 2015). In response to decitabine, increased CD80 expression was confirmed in CD80-negative cell lines, and the CD80 promoter was shown to be hypomethylated (Wang et al., 2013). DNA methylation also makes the fixation of T cell lineage identity. For example, differentiated Th2 cells lacking DNMT3a could easily induce IFN-γ production once placed in Th1 conditions, whereas wild-type cells remained much more stable in their lineage decision (Gamper et al., 2009). Azacitidine on CD4^+^ T-cells *in vitro* reduced proliferative capacity, reduced suppressor function, and increased IL-17 production. In a separate cohort of MDS patients, the effect of azacitidine appeared to transiently increase Treg cells and decrease Th17 population *in vivo* (Bontkes et al., 2012). Although Treg activation in response to decitabine led to increased IFN-γ expression and induction of a Th1 phenotype, the Treg maintained its suppressive capacity (Landman et al., 2018). Importantly, some clinical studies and observations also suggest the immune effects of HMAs after allo-HSCT. The effects of T-cell subsets by HMAs for three relapsed AML patients post-allo-HSCT has been reported: HMAs increased the populations of CD4^+^CD25^+^FOXP3^+^ regulatory T-cells (also confirmed as CD4+CD25hiCD127lo T-cells); HMAs also decreased CD8^+^ T-cells and Th1 cells, decreased CD8^+^ cytotoxicity, decreased IFN-γ production, and increased the transcription of IL-10 and TGF-β cytokines (Stübig et al., 2014). In 28 patients post-transplantation (16 patients with relapse), an increase of CD8^+^ T cell lineage was associated with a reduced risk of disease relapse and improved relapse-free survival (Craddock et al., 2016b).

Several studies have reported that chidamide in combination with decitabine exhibited a synergistic effect against leukemia cells *in vitro* and *in vivo*. A research for HL60 and NB4 cell lines showed that chidamide combined with decitabine synergistically inhibited proliferation with dose- and time-dependent blocked cell cycles by the induction of p21 expression and induced cell apoptosis by up-regulation of Bax and Caspase-3 and down-regulation of Bcl-2 (Mao et al., 2018). Another research also suggest that low-dose DAC and chidamide have synergistic anti-leukemia effect on K562 and THP-1 cell lines (Xu et al., 2019). Li et al. confirmed the synergistic effect of chidamide and decitabine on the AML cell lines (THP-1, MV4-11, HL60, and Kasumi-1) and primary cells of relapsed/refractory AML. They further demonstrate, for the first time, the role of PERP in the response of AML to a combination drug regimen, providing a new potential treatment protocol and target (Li et al., 2020). Our clinical practice by patients post-transplantation showed that the double-epigenetic regimen, including chidamide combined with decitabine, has surprising outcomes, which is the strongest evidence for a synergistic mechanism.

Interestingly, we observed that Th17 cells were increased, while Th1 and CTL cells were markedly decreased in patients with MRD relapse. How do we explain this observation? According to Gérard Eberl's review (Eberl, 2016), the immune system relies on an equilibrium in four different types of immune response (type 1 by Th1, type 2 by Th2, type 3 by Th17, and type 4 by TGFβ, with Tregs in these types): the equilibrium is defined as homeostasis. In those patients with MRD relapse, the equilibrium of Th cells is in chaos and benefited for the growth of leukemia cells. This dysregulation may be due to the tumor-mediated immune microenvironment, characterized by the Th17 population increasing and Th1 population decreasing. Importantly, this dynamic equilibrium appears not only in macroscopical level but also in microcosmic level. Lineage commitment not only entails that cells acquire the capacity to express lineage-specific factors but also that they silence signature genes defining other lineages. The differentiated phenotype requires the acquisition of a degree of stability (that is, the ability to “remember” a given phenotype) while at the same time maintaining some flexibility to respond to the changing environment. Epigenetic mechanisms, including post-translational modifications of histone tails and DNA methylation, play crucial roles in regulating all aspects of T cell differentiation, including the stability of the newly acquired phenotype (Avni et al., 2002; Monticelli, 2019). Further mechanistic studies will undoubtedly need to shed light on this issue.

In a word, the epigenetic regimen is safe and tolerable in non-APL AML patients who have post-transplant relapse. Although limited by sample size, our study showed the promising clinical efficacy of this regimen. Therefore, it represents promising alternatives or adjuncts to current treatment modalities for patients with post-transplantation relapse, and the Th1/Th17 ratio seems to reflect the drug-mediated immune effect, which may be a valuable marker to be monitored for evaluating therapeutic effect and prognosis. However, our study is far from satisfactory due to the limited number of patients. This may lead to overoptimistic outcomes than thought. Nevertheless, with the increasing cases, the updating data which reveal the truth will be obtained. In addition, mechanism research needs to be completed as soon as possible.

## Data Availability Statement

The original contributions presented in the study are included in the article/supplementary materials, further inquiries can be directed to the corresponding author.

## Ethics Statement

The studies involving human participants were reviewed and approved by the Ethics Committee of Affliated Hospital of University of Electronic Science and Technology of China. Written informed consent to participate in this study was provided by the participants' legal guardian/next of kin.

## Author Contributions

The manuscript was written by YX and DJ. The specimens was provided by LC and ZR. At last, the article was modified by ZH, WX, WC, and HX. All authors contributed to the article and approved the submitted version.

## Conflict of Interest

The authors declare that the research was conducted in the absence of any commercial or financial relationships that could be construed as a potential conflict of interest.

## Publisher's Note

All claims expressed in this article are solely those of the authors and do not necessarily represent those of their affiliated organizations, or those of the publisher, the editors and the reviewers. Any product that may be evaluated in this article, or claim that may be made by its manufacturer, is not guaranteed or endorsed by the publisher.
